# Antileishmanial Activity of 2-Methoxy-4H-spiro-[naphthalene-1,2′-oxiran]-4-one (Epoxymethoxy-lawsone): A Promising New Drug Candidate for Leishmaniasis Treatment

**DOI:** 10.3390/molecules23040864

**Published:** 2018-04-10

**Authors:** Luiz Filipe Gonçalves Oliveira, Franklin Souza-Silva, Luzia Monteiro de Castro Côrtes, Lea Cysne-Finkelstein, Mirian Cláudia de Souza Pereira, Francisco Odêncio de Oliveira Junior, Rosa Teixeira Pinho, Suzana Corte Real, Saulo Cabral Bourguignon, Vitor Francisco Ferreira, Carlos Roberto Alves

**Affiliations:** 1 Laboratório de Biologia Molecular e Doenças Endêmicas, Instituto Oswaldo Cruz-Fundação Oswaldo Cruz, Avenida Brasil, 4365, Manguinhos, Rio de Janeiro 21040-900, RJ, Brasil; luizfilipeol07@gmail.com (L.F.G.O.); fsilva@ioc.fiocruz.br (F.S.-S.); lmccortes@gmail.com (L.M.d.C.C.); 2 Laboratório de Imunoparasitologia, Instituto Oswaldo Cruz-Fundação Oswaldo Cruz, Avenida Brasil, 4365, Manguinhos, Rio de Janeiro 21040-900, RJ, Brasil; lcysne@ioc.fiocruz.br; 3 Laboratório de Ultraestrutura Celular, Instituto Oswaldo Cruz-Fundação Oswaldo Cruz, Avenida Brasil, 4365, Manguinhos, Rio de Janeiro 21040-900, RJ, Brasil; mirian@ioc.fiocruz.br (M.C.d.S.P.); forj@ioc.fiocruz.br (F.O.d.O.J.); 4 Laboratório de Imunologia Clínica, Instituto Oswaldo Cruz-Fundação Oswaldo Cruz, Avenida Brasil, 4365, Manguinhos, Rio de Janeiro 21040-900, RJ, Brasil; rospinho@ioc.fiocruz.br; 5 Laboratório de Biologia Estrutural, Instituto Oswaldo Cruz-Fundação Oswaldo Cruz, Avenida Brasil 4365, Rio de Janeiro 21040-900, RJ, Brasil; scr@ioc.fiocruz.br; 6 Laboratório de Interação Celular e Molecular-LICEM, Departamento de Biologia Celular e Molecular, Universidade Federal Fluminense, Niterói 24020-141, RJ, Brazil; saulo@vm.uff.br; 7 Departamento de Tecnologia Farmacêutica, Faculdade de Farmácia, Universidade Federal Fluminense, Niterói 24241-002, RJ, Brasil; cegvito@vm.uff.br

**Keywords:** antileishmanial activity, oxiranes, naphthoquinones, epoxymethoxy-lawsone, meglumine antimoniate

## Abstract

Epoxymethoxy-lawsone is a naphthoquinone derivative promising as drug candidate for the treatment of leishmaniases. In the present work the effectiveness of epoxymethoxylawsone, and meglumine antimoniate on *Leishmania* (*Leishmania*) *amazonensis* parasites and on mice paw lesions of infected BALB/c mice was assessed. In an intracellular amastigotes assay, the half-maximal inhibitory concentration (IC_50_) value for epoxymethoxylawsone was slightly higher (1.7-fold) than that found for meglumine antimoniate. The efficacy of both drugs became more evident after 48 h of exposure when either the oxirane compound and reference drug reached 18-fold and 7.4-fold lower IC_50_ values (0.40 ± 0.001 µM and 0.60 ± 0.02 µM), respectively. Promastigotes were also affected by epoxymethoxylawsone after 24 h of incubation (IC_50_ = 45.45 ± 5.0 µM), but with IC_50_ 6-fold higher than those found for intracellular amastigotes. Cytotoxicity analysis revealed that epoxymethoxylawsone (CC_50_ = 40.05 ± µM) has 1.7-fold higher effects than meglumine antimoniate (CC_50_ = 24.14 ± 2.6 µM). Treatment of the paw lesion in infected BALB/c mice with epoxymethoxy-lawsone led to a significant 27% reduction (*p* < 0.05) of the lesion size, for all administrated doses, compared to the control group. Lesion reduction was also detected after mice treatment with meglumine antimoniate, reaching 31.0% (0.23 mg of Sb(V)/Kg/day and 2.27 mg of Sb(V)/Kg/day) and 64.0% (22.7 mg of Sb(V)/Kg/day). In addition, mice lesion ultrastructural changes were evidenced in amastigotes. The set of data gathered here indicate that epoxymethoxylawsone has pronounced effects on parasites and merits furthering to the preclinical stage.

## 1. Introduction

Leishmaniasis is classified as one of the neglected tropical diseases by the World Health Organization which estimates that 350 million people are at risk of contracting this infection, while nearly two million new cases occur annually [1]. The infection is caused by more than 20 *Leishmania* species, which are transmitted by inoculation of promastigote forms in humans through the bite of infected female phlebotomine sandflies. In the mammalian host, these parasites differentiate into amastigote forms inside cells and affect skin, mucosa, and cartilage, causing cutaneous leishmaniasis (CL). However; some species can infect internal tissues and organs, such as the liver, spleen, and bone marrow, causing visceral leishmaniasis (VL). Mucosal leishmaniasis (ML) is a metastatic outcome of the cutaneous form in which the parasites become disseminated to the oropharyngeal mucosa [2]. The epidemiology of leishmaniasis depends on the characteristics of the parasite species, the ecological features of the transmission sites, and the degree of current or past exposure of the population to the parasite. Furthermore, the risk factors of transmission are linked to socioeconomic and environmental patterns, which can make disease control more difficult [3].

Despite some important recent advances in the diagnosis, treatment and cost reduction of key drugs, both mortality and morbidity show a worrying increasing trend worldwide. This can be attributed to several factors, including lack of a vaccine, ineffective vector control and limitations of current drugs used to treat the infection [4,5]. 

**Figure 1:**
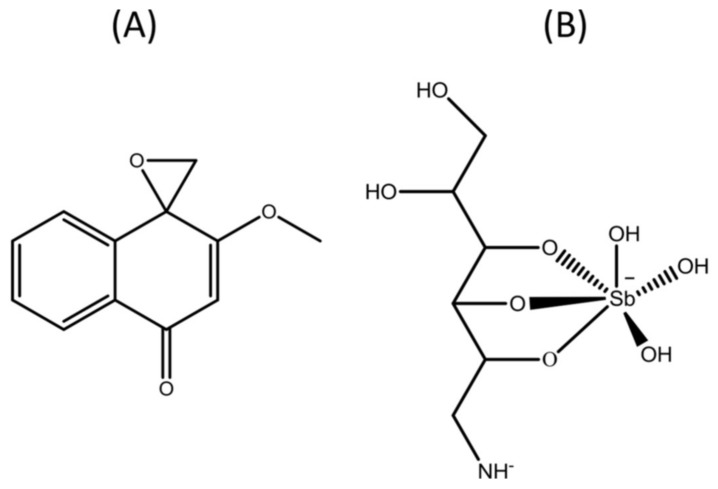
Chemical structure of drugs. (**A**) 2-methoxy-4H-spiro[naphthalene-1,2′-oxiran]-4-one, also known as epoxymethoxylawsone (C_12_H_10_O_3_, 202.21 g/mol) and (**B**) meglumine antimoniate known commercially as Glucantime^®^ (C_7_H_18_NO_8_Sb, 365.98 g/mol—structure proposed by Frézard et al. [15]).

Pentavalent antimonials, such as meglumine antimoniate (Figure 1), have been used since the 1940s and remain the first-choice drugs to treat all clinical forms of leishmaniasis, due to the even higher risks of toxicity associated with the second line drugs amphotericin B and pentamidine. These second line drugs are only used when there is a contraindication, intolerability or resistance to the first line drugs [6]. Nevertheless, pentavalent antimonials are frequently associated with high frequencies of mild to severe adverse effects, including musculoskeletal pain, gastrointestinal disorders, headache and anorexia, as well as cardiac, hepatic and pancreatic toxicity, leading in some cases to death [7]. At the same time, the large pharmaceutical companies have made little investment in research to develop therapeutic alternatives for leishmaniasis which explains the paucity of compounds and formulations with low toxicity and proven effectiveness in clinical use.

Consequently, the search for plant products is gaining special attention because they are theoretically more accessible, usually cheap and can be made accessible to lower income population who are the most affected by the disease [8]. A variety of natural products obtained from plant extracts has proved to be active against *Leishmania* species. Among these, the 1,4-naphthoquinones are considered attractive structures in medicinal chemistry due to their biological activities and chemical properties [9]. Examples of 1,4-naphthoquinones that have shown activity against *Leishmania* species and *Trypanosoma cruzi* are lapachol, isolated from Brazilian trees belonging to the genus *Tabebuia* and its derivatives α-lapachone and β-lapachone [10]. Most of the lapachone derivatives however, exhibit significant toxicity that limits their potential as new drugs [11]. 

In a search for less toxic derivatives for mammalian cells, chemical modification of the quinonoid center of α-lapachone and 2-hydroxy-1,4-naphthoquinone (lawsone) followed by an epoxidation, generated the oxiranes epoxy-α-lapachone and epoxymethoxylawsone (Figure 1), respectively [12]. We have demonstrated that epoxy-α-lapachone was capable to kill promastigote forms of *Leishmania* (*Viannia*) *braziliensis* and *Leishmania* (*Leishmania*) *amazonensis* and intracellular amastigotes in human macrophages [13]. Furthermore, reduction of the lesion size in the paw of BALB/c mice infected was observed after four weeks of treatment [14]. 

Previous data showed that epoxymethoxylawsone has a significant effect on control of BALB/c mice paw lesion caused by *L.* (*L.*) *amazonensis* [16]. In the present study, unequivocal evidence is presented of the antileishmanial activity of this oxirane compound on intracellular amastigotes and promastigote forms as well as in the control of the paw lesion caused by *L.* (*L.*) *amazonensis*. 

## 2. Results 

### 2.1. Drug Effects against Intracellular Amastigotes and Promastigotes of Leishmania (L.) amazonensis 

**Figure 2:**
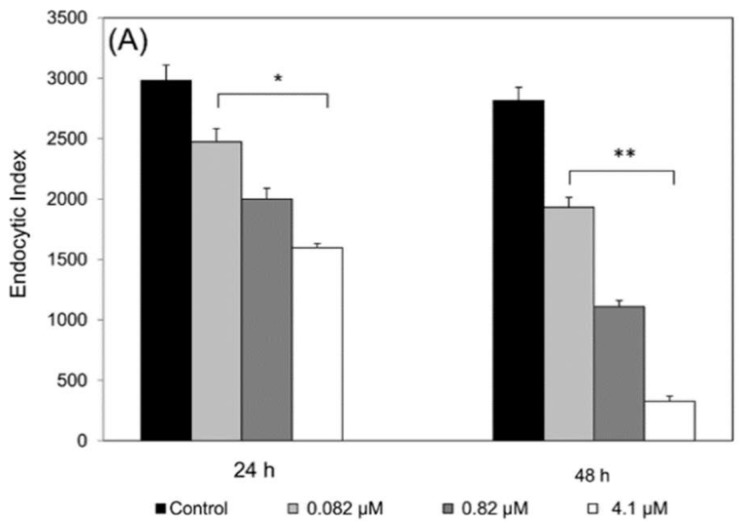

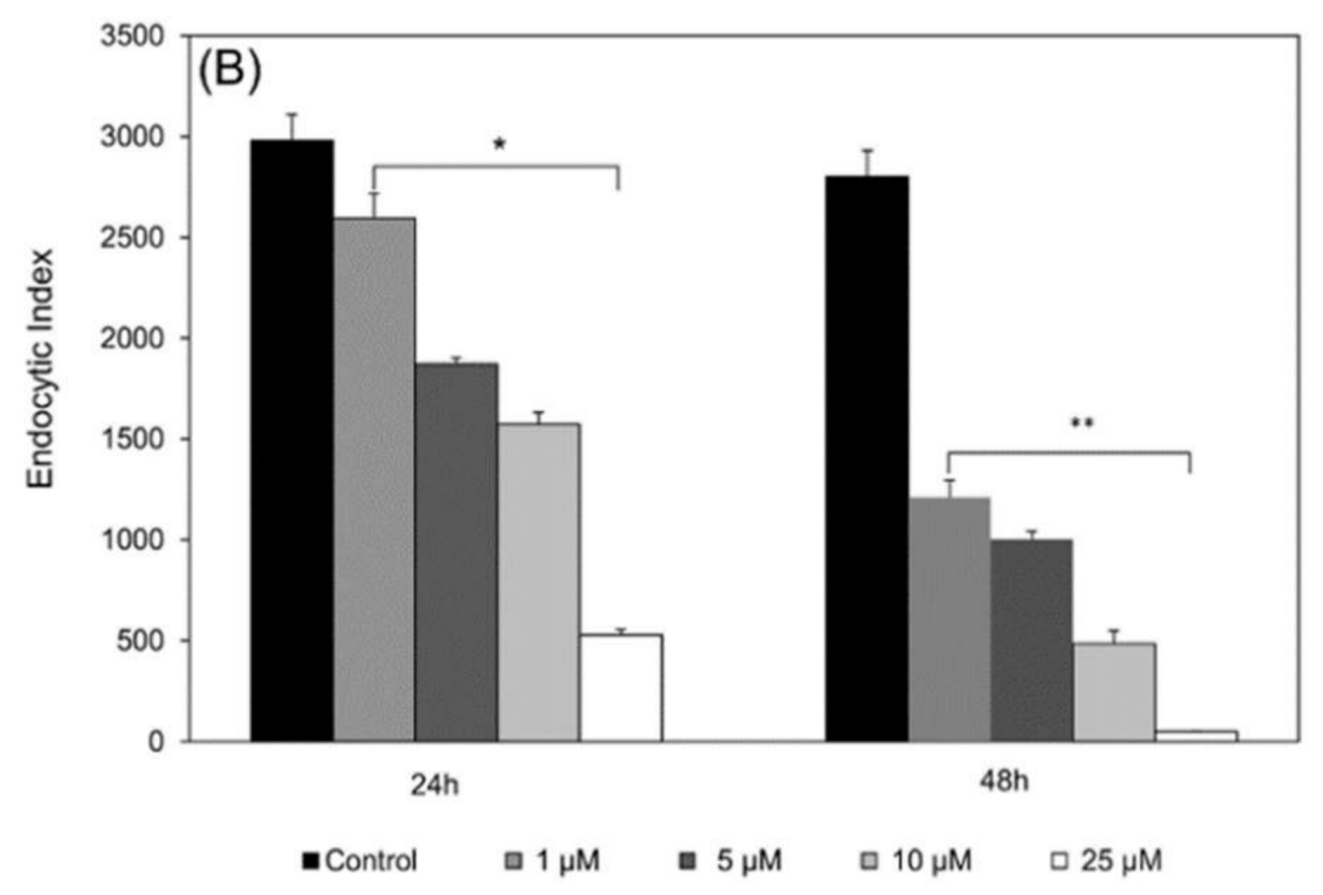
Effects of the drugs on the endocytic index of the *Leishmania* (*L*.) *amazonensis* amastigotes in mice macrophages. Meglumine antimoniate (**A**) and epoxymethoxy-lawsone (**B**) were co-incubated in cultures of BALB/c mice macrophages infected with *L.* (*L.*) *amazonensis* for 24 h and 48 h. Control cultures (black bars) were treated with RPMI 1640 medium only or with 1% of DMSO, respectively. The results are expressed as the mean and standard deviation of three assays. All concentration points analyzed showed statistical significance from their respective controls: (*) *p* ≤ 0.042; (**) *p* ≤ 0.009; (***) *p* ≤ 0.0003).

BALB/c mice macrophages infected with *L.* (*L.*) *amazonensis* treated with epoxymethoxylawsone showed a significant decrease in the number of viable parasites compared to control cultures. Reference drug meglumine antimoniate also exhibited significant effects. Both drugs were able to kill intracellular amastigotes in a dose-dependent manner at 24 and 48 h of exposure (Figure 2). All concentrations tested resulted in endocytic index (EI) lower than the control groups incubated with medium or 0.8% DMSO (Figure 2). The inhibitory effect on the multiplication rate at the highest concentration of epoxymethoxy-lawsone (25 µM) was 82.2% and 98.3% at 24 and 48 h, compared to the controls EI = 2980 and EI = 2816, respectively. Meglumine antimoniate (4.1 µM) also caused a pronounced inhibitory effect on the parasite multiplication (46% and 88.4% at 24 and 48 h, respectively). Half-maximal inhibitory concentration (IC_50_) determined for epoxymethoxy-lawsone in 24 h was about 18-fold higher than 48 h, while for meglumine antimoniate the difference between two incubating times was 7.4-fold (Table 1). To evaluate possible macrophage activation by the drugs, we determined nitric oxide (NO) production in the cultures supernatants, however no significant change in the NO levels for both treatments were observed (0.9 ± 0.01 mM) compared to the control group (data not shown). 

Epoxymethoxy-lawsone also inhibited the growth of free-living promastigote and the IC_50_ determined for 24 h was six-fold higher than that found for intracellular amastigotes, suggesting that the oxirane compound has a stage-specific effect. Table 1 presents the selectivity and specificity indexes for two drugs. Selectivity values for both drugs were very similar (>5.00), indicating that the toxic concentration for a mammalian cell (in this case murine macrophages) is five-fold higher than those toxic to the parasite. Additionally, we have assessed the effects of epoxymethoxylawsone in human macrophages infected by *L.* (*L.*) *amazonensis* (data not shown). In these assays, the drug at concentrations of 1 µM and 10 µM caused a reduction of 65.4% and 87.9%, respectively, when compared with control (0.8% DMSO).

### 2.2. Effects In Vivo of Treatment on Experimental Cutaneous Lesions Caused by Leishmania (L.) amazonensis

**Figure 3:**
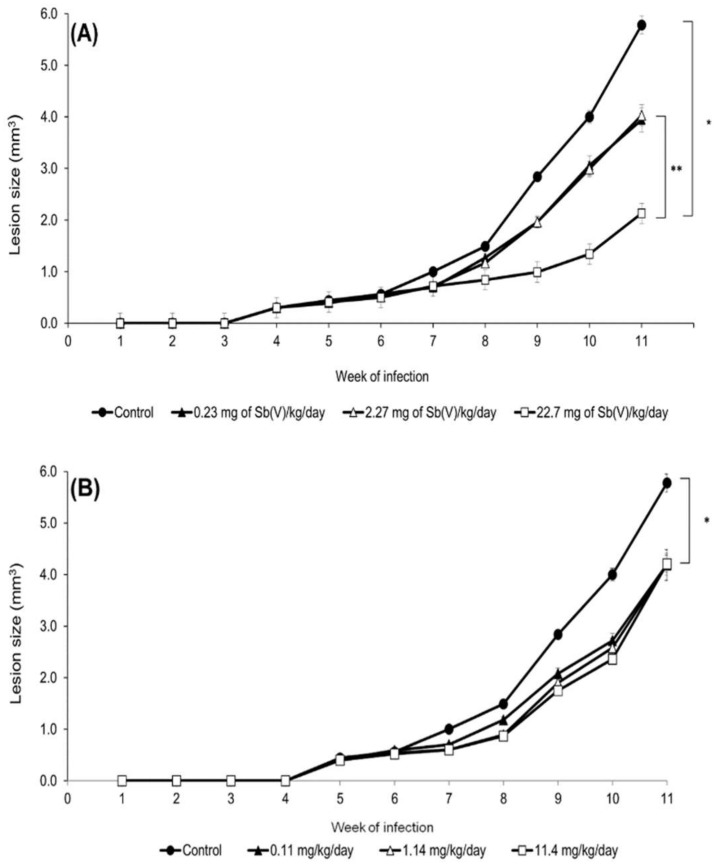
Treatment of experimental infection in BALB/c mice caused by *Leishmania* (*L.*) *amazonensis*. Mice were inoculated subcutaneously, in the left footpad, with 1.0 × 10^4^ promastigotes at the logarithmic phase of growth. After 4 weeks of infection, mice were treated daily with meglumine antimoniate (**A**) or epoxymethoxylawsone (**B**) at three different concentrations administered subcutaneously in groups with five animals. The control group was treated with a mix of DMSO/propylene glycol/saline (1:9:10). The lesion sizes were measured weekly and the results are represented as means with standard deviations from three independent experiments. Analyzed points exhibited significant differences from the control, (*) *p* ≤ 0.03, and with the groups, (**) *p* ≤ 0.02.

BALB/c mice treated with three different concentrations of epoxymethoxylawsone and meglumine antimoniate showed significantly reductions in the lesion size after 4 weeks of treatment compared to the control group (Figure 3). Maximum lesion sizes measure after 11 weeks post-infection was 5.8 ± 0.11 mm^3^ in control group treated with vehicle. Despite the fact no dose-response association was observed in the epoxymethoxylawsone treatment, the effects of the compound on lesion reductions (27.3%, 27.5% and 27.2% for 11.4 mg/Kg/day, 1.14 mg/Kg/day and 0.11 mg/Kg/day, respectively) were all statically significant of those detected in the vehicle groups (*p* < 0.03). Lesion reduction was also observed in mice groups which received the intermediate and lower doses of meglumine antimoniate, reaching to 31.0% (0.23 mg of Sb(V)/Kg/day and 2.27 mg of Sb(V)/Kg/day) of untreated group (*p* < 0.03). The highest lesion reduction value was observed in the mice group treated with meglumine antimoniate at a dose of 22.7 mg of Sb(V)/Kg/day, reaching 64% of the control value (*p* < 0.02).

### 2.3. Effects of the Treatments in BALB/c Skin Lesions by Light Microscopy and Transmission Electron Microscopy

**Figure 4:**
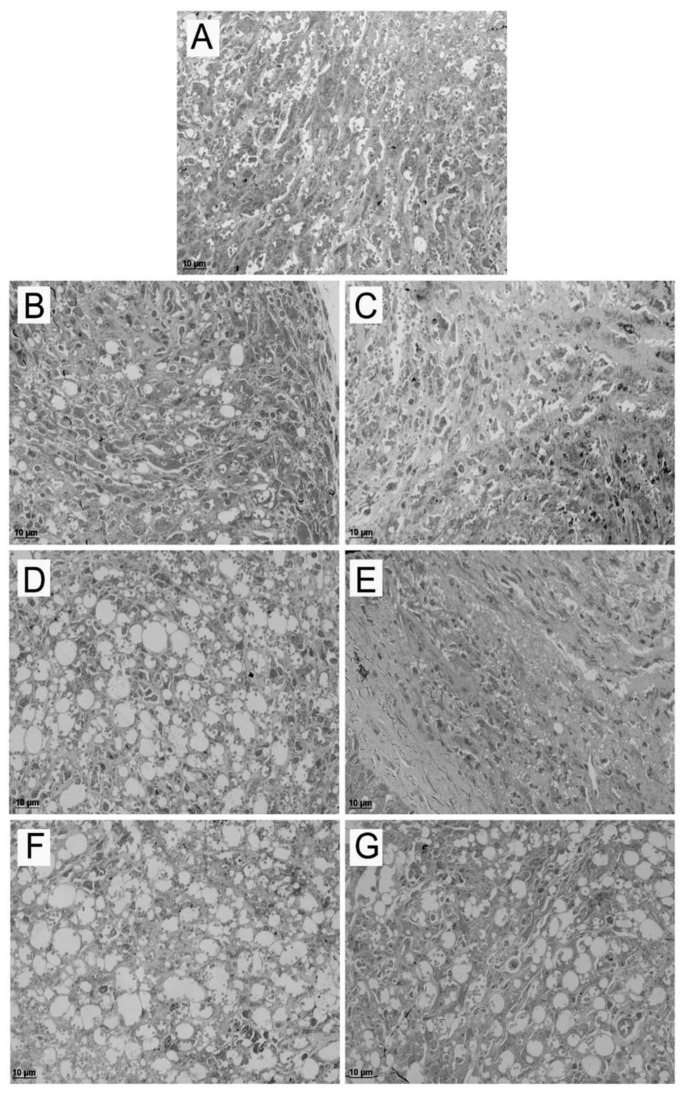
Analysis of mice lesions by light microscopy. Semithin sections of mice skin lesions of untreated (**A**) and treated groups with three doses of meglumine antimoniate (**B**: 22.7 mg of Sb(V)/kg/day; **D**: 2.27 mg of Sb(V)/kg/day; **F**: 0.23 mg of Sb(V)/kg/day) and epoxymethoxylawsone (**C**: 11.4 mg/kg/day; **E**: 1.14 mg/kg/day; **G**: 0.11 mg/kg/day). Lesions were extracted one week after the end of a four weeks treatment course. The images are representatives of ten fragments of each group mice.

The skin lesions of untreated and treated mice groups were examined regarding the presence of both amastigotes and parasitophorous vacuoles (PV) which are indicators of the infection. Semiquantitative analysis of the skin lesion fragments showed a dose-response effect in the reduction of parasite load and number of vacuoles for epoxymethoxylawsone treatment compared to untreated group, while mice treated with higher and intermediate doses of meglumine antimoniate exhibited no difference in the parasite load (Table 2 and Figure 4). In the lower doses, we have observed a similar quantity of vacuoles of those found in the untreated group; however, the parasite load was lower in the treated groups (Table 2).

**Figure 5:**
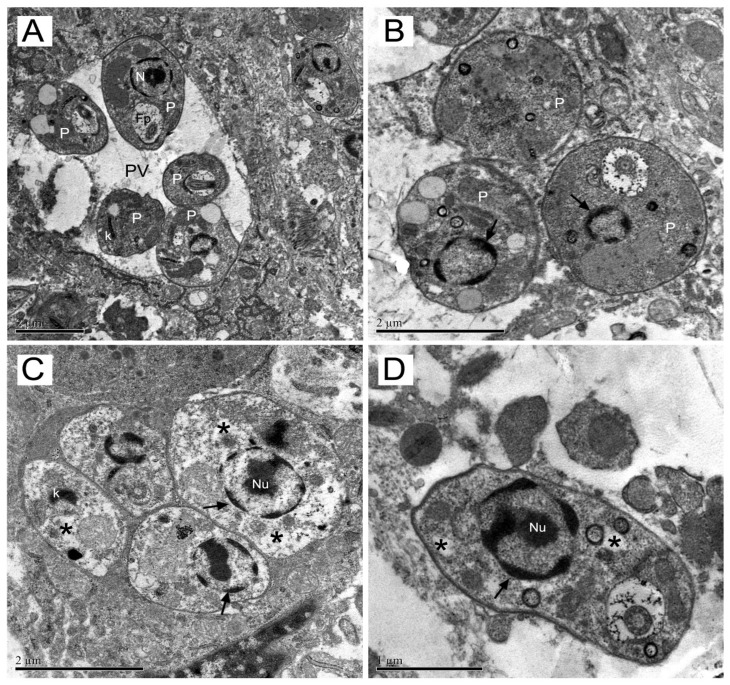
Transmission electron microscopy of mice skin lesion. The ultrastructural analysis of amastigotes from lesion were performed in untreated (**A**) or mice treated with meglumine antimoniate or epoxymethoxylawsone at higher doses (22.7 mg of Sb(V)/kg/day and 11.4 mg/kg/day, respectively). In (**A**): amastigotes with no morphological changes within parasitophorous vacuoles (PV); bar-shaped kinetoplast (K); nuclei (N) and flagellar pocket (Fp). In (**B**): Amastigotes (P) show dense nuclear chromatin (thin arrow) with altered profile and absence of nucleoli (→). In (**C**): Amastigotes (P) with rarefied cytoplasm (*); kinetoplast with an atypical condensation (k) and dense nuclear chromatin (→) and nucleolus (Nu). In (**D**): Amastigote (P) shows dense nuclear chromatin with altered profile and absence of nucleoli. The images are representatives of ten selections of each group mice.

Ultrastructural changes in amastigotes from skin mice lesion were assessed by transmission electron microscopy. The ultrastructural analysis of untreated amastigotes within parasitophorous vacuoles (PV) demonstrating their normal characteristics such as rounded or oval shape and cytoplasmic components as bar-shaped kinetoplast (K), nucleus (N) and flagellar pocket (Fp) with emerging flagellum (Figure 5A). Amastigotes from mice groups exposed to both drugs exhibited different degrees of damage. Amastigotes (P) from meglumine antimoniate group showed dense nuclear chromatin with an altered profile and, in most images analyzed, the nucleoli were not observed (Figure 5B). Mice treated with epoxymethoxylawsone, showed amastigotes from their lesions presenting rarefied cytoplasm (asterisk), kinetoplast with an atypical condensation (arrow head) and either a very dense chromatin (thin arrows) and nucleolus (Nu) (Figure 5C, D). Several of these ultrastructural alterations were also detected in the meglumine antimoniate treatment mice group (data not shown).

## 3. Discussion

Leishmaniasis remains as one of the most neglected tropical diseases, affecting mainly the poorer populations in developing countries. Current treatment, based on pentavalent antimony, is associated with severe side effects, including cardiotoxicity, hepatotoxicity and pancreatic toxicity [7]. Furthermore, high cost and technological dependence should be considered by endemic countries, increasing the need for new, more efficient and less toxic drugs. In this scenario, we highlight plant-derived compounds such as the naphthoquinones and its derivatives. Recently studies have demonstrated antileishmanial activity of epoxy-α-lapachone on macrophage infection and in the treatment of experimental murine infection with low cytotoxicity in mammalian cells [13]. Here, we tested epoxymethoxylawsone, a new oxirane derivative that also exhibited lower toxicity on mammalian cell against both in vitro and in vivo infection.

The approach proposed here to evaluate leishmanicidal effects of epoxymethoxylawsone was successful since we have proved its in vitro action on the multiplication rate of intracellular amastigotes and promastigotes growth, as well as in vivo ability to control the experimental mice paw lesion infection induced by *L.* (*L.*) *amazonensis*. In this study, we administrated meglumine antimoniate as reference control due to be a first-choice drug for all forms of human leishmaniasis treatment. Our results showed a similar selectivity index for both drugs, and their IC_50_ values were slightly different. Besides that, reference drug showed higher cytotoxicity on macrophages than oxirane compound (1.7-fold). Selectivity index is a key parameter to consider when developing antimicrobial compound and the higher reach this value the safer it will be, in theory, the therapeutic use of the drug, as exhibited for epoxymethoxylawsone here. 

Recently, a series of recommendations on criteria for selecting hit and lead compounds in drug discovery was published [17]. Selectivity values may vary considerably according to the microorganism and its environment in the host, but general recommendation is that this value should be greater than 10-fold using a mammalian cell line, as HepG2 or Vero cells [17]. Indeed, one of the most crucial elements for the success of drug screening is to apply assays that faithfully reproduce the microenvironment of a pathogen causing the disease. For this reason, we used murine macrophages due to this type of cell is the main target of *Leishmania spp* infection. Additionally, epoxymethoxylawsone demonstrated other relevant parameters for the *Leishmania* life cycle that is the specificity by the intracellular stage. It should be noted that epoxymethoxylawsone showed higher potency than epoxy-α-lapachone in infected macrophage cultures, represented by an IC_50_ value about five-fold lower for 24 h of exposure (7.41 μM compared to 37 μM), with similar cytotoxicity profile [13].

We have evidence that epoxymethoxylawsone reduced of lesion size in BALB/c mice paws due decreasing the parasite load, but not by reduction of the inflammatory process only, since was not possible to distinguish the lesion reduction effects among the three doses tested. In addition, this idea is reinforced by the observation that both epoxymethoxylawsone and meglumine antimoniate did not induce nitric oxide production in BALB/c mice macrophages infection. It is important to emphasize that drug efficacy measured solely by changes in lesion size can be misleading since a typical lesion is composed by a complex profile of inflammatory cells and amastigotes contained in vacuoles within macrophages of the skin [18].

In addition, the effects of drugs were perceived by the presence of vacuoles organelles with remaining parasite after elimination, mainly in mice groups treated with meglumine antimoniate as result of lesions treated by an effective drug. On the other hand, in the epoxymethoxylawsone groups, lesion tissues showed a lower number of vacuoles. These depleted organelles were extensively detected, indicating a drastic reduction of parasite load. 

To prove the drastic effect of treatments on the parasites from paw lesions, we decide to investigate ultrastructural alterations suffered by amastigotes to assess the integrity of parasites in skin tissue. Transmission electron microscopy analysis applied here revealed drastic changes in amastigotes ultrastructure which clearly affected parasite integrity. Epoxymethoxy-lawsone caused more pronounced changes than the reference drug.

Some of these alterations, such as those found in the amastigotes nuclei, are indicative of apoptosis events [19]. We are sure that those disorders found are in accordance with amastigotes inability to multiply in the macrophage mice lesions thus decreasing the paw lesion size in treated mice.

The data presented here suggest that epoxymethoxylawsone is capable of crossing the plasma membrane of the macrophages and acts by directly killing amastigotes, but the mode of action of the drug on *L.* (*L.*) *amazonensis* is not well known yet. As this compound is also derived from the α-lapachone molecule by an epoxidation reaction, and shares most of chemical and structural features with epoxy-α-lapachone, is possible that the new oxirane also acts by inhibiting proteases from *Leishmania* sp. and other trypanosomatids, as previously reported [12,14,20,21], however further studies are needed to prove this hypothesis.

## 4. Materials and Methods 

### 4.1. Chemicals and Culture Material 

Dimethyl sulfoxide (DMSO), osmium tetroxide solution (OsO_4_), Epoxy Embedding Medium kit (Epon), penicillin, streptomycin, Lab-Tek chamber slides, Greiner CELLSTAR^®^ 96 well plates, RPMI 1640 medium and Schneider’s Drosophila medium were purchased from Sigma-Aldrich Chemical Co. (St. Louis, MO, USA). Fetal calf serum (FCS) was acquired from Cultilab S/A (São Paulo, Brazil). CellTiter-Glo^®^ luminescent cell viability assay was acquired from Promega Corporation (Madison, WI, USA). Meglumine antimoniate (Glucantime^®^) was kindly provided by Dr. Armando de Oliveira Schubach team (INI/Fiocruz). Propylene glycol was obtained from Vetec Quimica (Rio de Janeiro, Brasil). The epoxymethoxylawsone compound was synthesized by the Department of Organic Chemistry of the Instituto de Química, Universidade Federal Fluminense and the powder was stored at 2 to 8 °C until its further use in assays.

### 4.2. Cell Culture

Peritoneal macrophages were harvested from BALB/c mice as previously described [22]. Cells were recovered after centrifugation (2 ×, 1800 × *g*, 10 min, 4 °C) in RPMI 1640 medium containing 10% FCS. Subsequently, cells were seeded at a density of 5 × 10^5^ cells/well in Lab-Tek chamber slides and maintained at 37 °C in a 5% of CO_2_ atmosphere for 24 h. Non-adherent cells were removed by washing the culture plates with RPMI 1640 medium. Monolayers of murine peritoneal macrophages were used in leshmanicidal assays.

### 4.3. Parasite Cultures 

*Leishmania* (*Leishmania*) *amazonensis* (strain MHOM/BR/73/LTB0016) was obtained from the *Leishmania* collection (Coleção de Leishmania do Instituto Oswaldo Cruz—CLIOC) of the Instituto Oswaldo Cruz (IOC). In vitro promastigote cultures were maintained at 28 °C in Schneider’s medium (pH 7.2) containing 1 mM l-glutamine, 10% FCS, 100 IU/mL penicillin, and 100 µg/mL streptomycin, with frequent subpassages to maintain the parasites in the logarithmic growth phase. 

### 4.4. Activity against Promastigotes 

Parasites were seeded on 96-well plates (1 × 10^5^ per well) in Schneider’s medium, were treated for 24 h at 28 °C with epoxymethoxylawsone in a concentration ranging from 1.6 µM to 100 µM. Parasite viability was assessed by measuring ATP production using CellTiter-Glo^®^ (50 µL/well) and the luminescent signal was measured using a FlexStation 3 reader (Molecular Devices, Sunnyvale, CA, USA) [23]. Drug efficacy determined by half maximal inhibitory concentration (IC_50_) was calculated by linear regression.

### 4.5. Activity against Intracellular Amastigotes 

To evaluate the activity of compounds against intracellular amastigotes, macrophages were infected by promastigotes in a proportion of 10:1 (parasite:cell) for 4 h of interaction, at 37 °C followed by washing with PBS and addition of RPMI medium containing 5% FCS. After 4 h of infection, the cultures were treated for 24 h or 48 h at 37 °C with epoxymethoxylawsone (1 to 25 µM) and meglumine antimoniate (0.082 to 4.1 µM) and then, fixed with 100% methanol and Giemsa-stained. The level of infection and number of intracellular parasites was determined by random counting of at least 300 cells. The endocytic index was calculated by multiplying the percentage of infected cells by the mean number of parasites per infected cell. Drug inhibition concentrations (IC_50_) were calculated by regression analysis of dose-response curves. Selectivity index (SI) was calculated by the following formula: SI = CC_50_ (macrophage cytotoxicity) divided by IC_50_ (antiparasitic activity) for both drugs. The experiments were carried out in triplicate.

### 4.6. Toxicity to Mammalian Cells 

BALB/c mice macrophages grown on 96-well plates were treated with epoxymethoxy-lawsone (1.6 to 100 µM) and meglumine antimoniate (4.1–200.0 µM of Sb^5+^) for 72 h at 37 °C. Then, macrophages viability was determined by incubation with CellTiter-Glo^®^ (20 μL/well) for 3 minutes at room temperature under agitation. Luminescence was measured using a FlexStation 3 reader (Molecular Devices). The CC_50_, concentration of compound that reduces 50% of mammalian cell viability was determined by linear regression. Control was incubated with dimethyl sulfoxide (DMSO) in concentrations ≤1%.

### 4.7. Experimental Murine Infection 

Experimental infection was conducted with 5- to 7-week-old BALB/c mice weighing approximately 22 g. Mice were inoculated in the footpad of the left hind limb with 1.0 × 10^4^ promastigotes of *L.* (*L.*) *amazonensis* in the stationary growth phase (after 5 days of culture in Schneider’s medium) in a total volume of 50 μL of phosphate-buffered saline (PBS) at 10 mM.

### 4.8. Mice Treatment Schedules 

The experimental treatments were performed with either antimoniate meglumine, as a comparative control for treatment efficacy and epoxymethoxylawsone diluted in a mixture of DMSO/propylene glycol/saline (1:9:10, defined as vehicle) in the following doses: 22.7 mg of Sb(V)Kg/day, 2.27 mg of Sb(V)/Kg/day and 0.23 mg of Sb(V)/Kg/day (corresponding to 4.1 µM, 0.41 µM and 0.041 µM of Sb(V)) and 11.4 mg/Kg/day, 1.14 mg/Kg/day and 0.11 mg/Kg/day (corresponding to 1.2 µM, 0.12 µM, and 0.012 µM of oxirane compound). Drugs were administered subcutaneously in the dorsal region of each mouse in a dose of 100 µL per animal. Treatments were carried out for four weeks with daily injections (five consecutive days with a two-day pause until 20 doses), starting four weeks after challenge infection, when the paw lesions had already become noticeable. Negative-control groups were treated with vehicle used to dissolve the oxirane compound. The lesions were evaluated weekly by measuring the height and width of the paw and calculating lesion areas (obtained by multiplying these measures in mm^2^) with a digital caliper.

### 4.9. Processing of Samples for Transmission Electron Microscopy 

Lesions were excised with a surgical scissors after 20 days of treatment (50 days post infection), washed in PBS and fixed in 2.5% glutaraldehyde in 0.1M sodium cacodylate buffer pH 7.2 with 3.5% sucrose for 1h/4 °C. Samples were post-fixed with 1% OsO_4_ (1 h/4 °C) in cacodylate buffer and, after washing, the samples were dehydrated in serial acetone concentrations (30%, 50%, 70%, 90% and 100%). Finally, samples were embedded in PolyBed 812 resin and polymerized at 72 h/ 60 °C. After polymerization, semi-thin sections were made in an Ultracut S ultramicrotome (Leica, Vienna, Austria) stained with toluidine blue and eosin and analyzed under a light microscope - Axio imager 2 (Zeiss, Göttingen, Germany). After selecting the areas of interest, ultra-thin sections were obtained in Leica Ultracut S ultramicrotome, collected in 300 mesh copper grids, contrasted with 5% and lead citrate and analyzed in the transmission electron microscope - JEOL JEM-1011 (Boston, MA, USA). 

### 4.10. Semiquantitative Analysis of Fragments 

All fragments were semiquantitatively assessed based on the intensity and focal or diffuse character of the infection, considering presence of vacuoles and quantity of amastigotes and the results were plotted as the media of both amastigote number and vacuoles in macrophages. For each fragment a parameter was assigned with a numerical value between + and ++++, according to the intensity and extent of the infection: + = mild, ++ = moderate, +++ = severe, and ++++ = severe-diffuse. The samples were analyzed under light microscope (Zeiss Axio imager m2).

### 4.11. Ethical Aspects 

Mice experimental procedures performed here were approved by the Committee for the Ethical Use of Animals of Instituto Oswaldo Cruz (L-052/2015). The animals were obtained from the animal breeding center of Fundação Oswaldo Cruz (Fiocruz). The additional assays with human cells from healthy donors were approved by the Committee of Ethics Fiocruz (C.E. Fiocruz protocol number 535/09). 

### 4.12. Statistical Analysis 

To compare results, Student’s test was applied; data matrices were considered statistically different when the P value was less than 0.05. Statistical analyses were performed using GraphPad Prism version 5.03 (GraphPad Software, San Diego, CA, USA).

## 5. Conclusions

The set of results gathered here prove that chemical modification made on the 2-hydroxy-1,4-naphthoquinone generated another effective and with low toxicity derivative from same chemical synthesis series of other oxirane compounds **12**. We are sure that epoxymethoxylawsone has reached the minimum requirements to advance to the preclinical stage, including in vitro and in vivo complementary tests.

## Figures and Tables

**Table 1:** Effects of drugs on the murine macrophage cells and on the *Leishmania* (*L.*) *amazonensis* parasites.

Drug	CC_50_ (μM)	IC_50_ (μM)			Selectivity *	Specificity **
	Murine macrophage	Promastigote	Intracellular amastigote			
	72 h	24 h	24 h	48 h		
Epoxymethoxy-lawsone	40.05 ± 3.0	45.45 ± 5.0	7.41 ± 0.2	0.40 ± 0.001	5.40	6.13
Meglumine antimoniate	24.14 ± 2.6	ND	4.43 ± 0.25	0.60 ± 0.02	5.45	ND

The values are expressed as concentration of drugs (µM) causing 50% of cellular cytotoxicity (CC) and inhibitory concentration of parasite multiplication, (IC) effects and represent the average and standard deviation (±) of three independent experiments. Data of murine macrophages CC_50_ and promastigotes IC_50_ were obtained by ATP-bioluminescence, and intracellular amastigote IC_50_ by endocytic index assays. Selectivity (*) is defined as the ratio between parasite IC_50_ and murine macrophages CC_50_. Specificity (**) is the ratio between promastigote IC_50_ and intracellular amastigote IC_50_. Specificity values higher than two were chosen to define a compound as more active against the intracellular amastigote stage.

**Table 2:** Semiquantitative analysis of BALB/c mice skin lesions caused by *Leishmania* (*L.*) *amazonensis.*

Mice Groups	Treatment Dose (mg/kg/Day)	Vacuoles	Amastigotes
Untreated	-	++++	++++
Epoxymethoxy-lawsone	11.4	+	+
	1.14	+	+
	0.11	++++	+++
Meglumine antimoniate	22.7	++	++
	2.27	+++	++
	0.23	++++	+++

(+) = mild, (++) = moderate, (+++) = severe, (++++) = severe-diffuse.
